# Adherence to artemether-lumefantrine drug combination: a rural community experience six years after change of malaria treatment policy in Tanzania

**DOI:** 10.1186/1475-2875-13-267

**Published:** 2014-07-10

**Authors:** Omary Minzi, Sylivester Maige, Philip Sasi, Billy Ngasala

**Affiliations:** 1 Unit of Pharmacology and Therapeutics, School of Pharmacy, Muhimbili University of Health and Allied Sciences, PO Box 65013, Dar Es Salaam, Tanzania; 2 Medical stores Department, PO Box 9081, Dar Es Salaam, Tanzania; 3 Department of Clinical Pharmacology, School of Medicine, Muhimbili University of Health and Allied Sciences, PO Box 65015, Dar Es Salaam, Tanzania; 4 Department of Parasitology, School of Social Sciences and Public Health, Muhimbili University of Health and Allied Sciences, PO Box 65012, Dar Es Salaam, Tanzania

**Keywords:** Adherence, Parasite clearance, Artemisinin-lumefantrine combination therapy

## Abstract

**Background:**

Adherence to multidosing is challenging worldwide. This study assessed the extent of adherence to multidosing artemether-lumefantrine (ALu) in a rural community in Tanzania, six years after switching from single dose policy of sulphadoxine-pyrimethamine.

**Methods:**

This study was a prospective observational, open label, non-randomized study involving 151 patients with uncomplicated malaria recruited at Fukayosi dispensary in Bagamoyo district in Tanzania. Patients treated with ALu were visited at home on day 3 for interview on drug intake, capillary blood sample collection for microscopy and ALu tablets count. Venous blood samples (2 ml) for determination of blood lumefantrine concentrations and blood slides for microscopy were collected on day-7. Kappa’s coefficient was used to assess agreement between pill count and self-report. Adherence was categorized depending on the tablets remaining and what the patient reported. Only those with empty blister pack available but no tablet remaining and reported taking all six doses of ALu at a correct dose and correct time were regarded as definite adherent. The rest were either probable adherent or probable non-adherent.

**Results:**

Only 14.9% of the patients were definite adherent the rest took the drug at incorrect time or did not finish the tablets. Out of 90 patients with analysed plasma samples for lumefantrine blood concentrations, 13/90 (14.4.0%) had lumefantrine concentrations <175 ng/ml. There was no difference in mean lumefantrine concentration in the patients who stated to have taken all doses as required (561.61 ng/ml 95% CI = 419.81-703.41) compared to those who stated to have not adhered well to drug intake (490.95 ng/ml, 95% CI = 404.18-577.7074 (p = 0.643). None of the patients had detectable parasites by microscopy on day-3 and day-7 regardless of adherence status and the level of day-7 blood lumefantrine. There was strong agreement between the self-reported responses on drug intake and pill-counts (kappa coefficient = 0.955). Age, sex, education and place where first dose was taken were associated with adherence.

**Conclusions:**

The overall adherence six years after the change of malaria treatment policy was low. It is, therefore, important to continuously monitor the level of adherence to treatment in order to get the current situation and institute corrective measures on time.

## Background

Adherence to multi-dosing regimen is a global challenge facing both developed and developing countries [1,2]. Adherence to long-term therapies in the general population is around 50% and is much lower in developing countries. Poor adherence to the prescribed treatment regimen leads to sub-curative doses and increases the rate of treatment failures. It also contributes to the emergence of drug resistance [3]. Due to drug resistance against chloroquine (CQ) and sulphadoxine-pyrimethamine (SP), many countries in sub-Saharan Africa, including Tanzania, changed the treatment policy of non-severe malaria to artemether-lumefantrine (ALu) drug combination [4]. However, for a successful treatment outcome, maximum adherence to this drug combination is necessary.

Since the introduction of ALu, a number of studies which assessed the extent of adherence have been published [5-12]. Assessment of adherence to medication is a multifaceted approach involving different subjective and objective methods. Adherence can be measured by using patient self-reporting, pill count and recording of the time for pill swallowing. Clinical attendances and other biological markers have been used for long-term chronic illnesses [1,13]. However, assessment of adherence in a short-term disease, such as malaria, may be challenging. During malaria treatment, the patient takes medication for not more than three days, and also determination of parasite clearance as marker of adherence may give false negative results since parasite clearance can be achieved even with incomplete dosing regimen [2]. Lastly, malaria being a short-term disease, does not involve several clinic attendances, hence excluding the use of number of missed clinic appointments as one of the methods for adherence assessment.

Several studies have demonstrated the usefulness of drug plasma concentrations in the assessment of adherence to drug intake [2,5,10,14]. Day-7 lumefantrine plasma concentrations is associated with malaria cure rate in patients taking a quality drug and adhering well to ALu and could be used as a complementary alternative in the assessment adherence to malaria treatment [2].

Studies have shown that adherence trends vary from the time the treatment policy was introduced. Fogg *et al.*[5] determined adherence to ALu in a semi-urban district in Uganda during the early stages of ALu policy implementation and obtained high adherence rates as opposed to the findings of Simba *et al.* in Tanzania two years after the new treatment policy was rolled out [10]. This study reports the extent of adherence to ALu treatment in a rural community in Tanzania, six years after adoption of a new malaria policy.

## Methods

### Study design

This was a prospective observational, non-randomized study conducted at a dispensary and household levels. The methods used in data collection were patient/caretaker self report, pill count, microscopy and determination of day-7 blood lumefantrine concentrations. The study was conducted between January and July, 2012.

### Study area and site

The study was conducted at Fukayosi dispensary and involved catchment villages. The dispensary is situated at Bagamoyo district in the Coastal region. The prevalence of malaria in children under five years of age in Coastal region is about 21%. In the rural area in Tanzania, there are administrative arrangements in which several villages form the so-called ward and several wards form a hamlet. Fukayosi ward is administratively comprised of five villages, namely Fukayosi, Kidomole, Mwavi, Msinune and Mkenge.

The study area has moderate perennial malaria transmission with higher sporozoite infection rates within the *Anopheles gambiae* complex ranging from 2% to 25%, with the peaks in January and July following the two rainy periods. Malaria disease is almost entirely due to *Plasmodium falciparum*, with *Plasmodium malariae* and *Plasmodium ovale* occurring in less than 5% of infections (often mixed with *P. falciparum*); *Plasmodium vivax* is rarely found. The main vectors are *An. gambiae sensu stricto*, *Anopheles arabiensis* and *Anopheles. funestus*[15].

### Study population

#### ***Sample selection and estimation of the sample size of the respondents***

Simba *et al.*[10] reported adherence rate to ALu treatment of 80% in a rural setting in Morogoro, Tanzania. Based on this, an assumption was made in which the proportion of the population adherent to ALu was 80%. The significance was set at p < 0.05 and the calculated sample size was N = 246 patients. Considering a maximum attrition rate of 15% and seasonal variation in parasitaemia among the participants, a total of 283 participants were recruited in the study.

### Patient recruitment

Patients were recruited on daily basis whenever they presented to the dispensary seeking for medical attention. A checklist was used to record the patient information, which was then analysed to decide the eligibility of the patient to participate in the study based on the inclusion and exclusion criteria.

### Inclusion criteria

Patients were included in the study if the met the following criteria: (i) Living within a 10 km perimeter from the dispensary, (ii) Adult male or female respondents, (iii) Caretakers having children six months and older, (iv) Children above 5 kg of weight; (v) Subjects confirmed with malaria; (vi) Subjects prescribed with ALu.

### Exclusion criteria

Patients were excluded from the study if one of the following criteria were found: (i) Female subjects in first trimester of pregnancy, (ii) Patients with known hypersensitivity to artemether and lumefantrine, (iii) Patients with severe malaria, (iv) Patients taking drugs which could interfere with the metabolic pathways of lumefantrine or drugs that are known to prolong QT intervals, such as antiarrythmics of Class IA and III.

### Patients recruitment

On the day of patient recruitment all patients followed the routine process at the dispensary such as consultation with the Clinical Officer, laboratory examination and received medications for their illness. The eligible patients also underwent through the same process and only those who were found to be malaria infected by microscopy and also had no previously documented allergy to ALu were included in the study. The patients were not informed that they would be followed at home on day 3 for assessment of adherence. On day-3, the patients were followed at home and were initially asked to give a written informed consent/assent. They were then interviewed on the drug intake and the remaining ALu tablets were checked and counted. They were requested to return to the dispensary on day-7 for collection of blood samples for microscopy and also sampling of blood for determination of blood lumefantrine concentration.

### Study procedures

**Figure 1:**
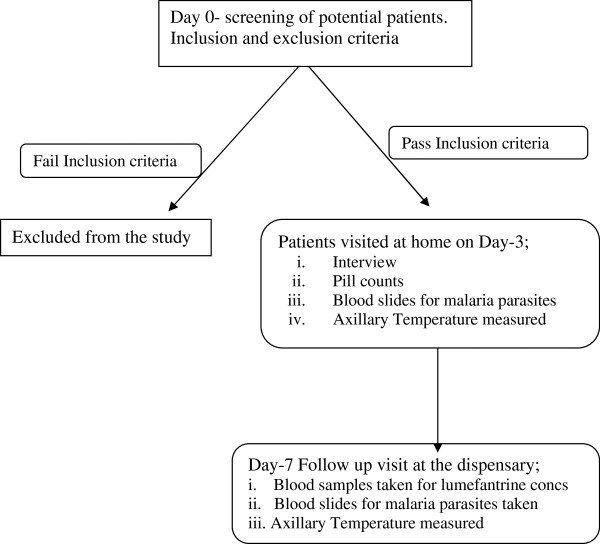
Flow chart for study procedures.

The patients followed the routine procedures adopted at Fukayose dispensary. The flow of patients is illustrated in Figure 1.

### Day-0 patients screening and drug administration

Day 0 corresponds to the day when the patient visited the dispensary. The patients were screened at the dispensary for presence of malaria by microscopy and using an algorithm for clinical diagnosis described in the guidelines of The National Malaria Control Programme. All patients found to be malaria positive were prescribed ALu and had to start ALu treatment regimen on the same day. As per the instructions from the manufacturer of ALu (Coartem^®^) (Novartis Pharma AG, Basel, Switzerland), ALu is dosed according to body weight: 5 to <15 kg, one tablet per dose; 15 to <25 kg, two tablets per dose; 25 to <35 kg, three tablets per dose; ≥35 kg, four tablets per dose. The first two doses of ALu are to be given eight hours apart on day 1. On days 2 and 3, ALu is to be given twice daily, 12 hours apart; with the morning dose being administered 24 hours after the first dose was taken.

The clinical examination was conducted by a clinical officer, whereas microscopy was conducted by a well-trained and highly experienced laboratory technician from Ifakara Health Institute. Slides were stained with Giemsa and read for parasitaemia by the same technician. Parasitaemia was calculated against 200 leukocytes according to the formula: parasitaemia (/μL) = number of parasites × 8,000/number of leukocytes. A slide was considered negative after 200 high-power fields had been examined. Those patients confirmed by microscopy as having malaria were prescribed ALu. The drug was dispensed by a trained nurse who gave standardized instructions to the patients on how to take ALu, with much emphasize on the correct time for taking ALu tablets and the need to take the drug with a fatty meal. Lastly, the nurse wrote down, in a standard form, the patient information, including date and time of consultation, age, sex, weight, name of the patient/caretaker, name of the head of household, the physical address (distance from the dispensary, name of the village and village leader) and phone number wherever possible. The patients were not informed that they will be visited at home on day-3 for assessment of medication intake.

### Day-3 home visits

A field team consisting of trained research assistants traced the patients’ homes on the day after the ALu regimen was supposed to have been completed (day-3). Before administration of the questionnaire a written informed consent and assent were sought from the eligible patients and from caregivers if the patient was less than 18 years old. The respondents and/or caretakers of eligible children in each of the homestead visited were then administered with a pre-piloted structured questionnaire. All questionnaire were administered in Swahili language by a trained research assistant to facilitate understanding. Respondents were patients themselves if ≥15 years old or their caretakers if younger. A structured interview to determine how the regimen was taken, the time and method of taking each dose was then conducted. Basic socio-demographic information was also collected (household size, level of education of respondent, number of children cared for by the same caretaker, marital status). Only one respondent or caretaker was interviewed from each of the household found to contain more than one participant. In the event where the respondent/caretaker was not at home on the day of home visit, the field team would trace and interview him/her if within a 10 kilometres perimeter.

### Uninformed home visit for pill-check and pill count

On day-3 blister pack check and pill count was performed by using a checklist. Availability of the blister pack, availability of unused ALu tablets and the number of ALu tablets remaining were assessed. The blister pack check combined with self-reported adherence enabled to classify the respondents according to category of adherence using categorization which had been adopted by another study [5].

### Categorization of adherence based on pill-check and patient self-reported method

Categories of adherence were classified as adherent or non-adherent as described below.

#### ***Non-adherent patients***

Those with unfinished tablets on Day-3 were categorized as definitely non-adherent and those who with empty blister pack available/missing, but reported taking ALu at a wrong dose were probably non-adherent. On the other hand, a patient who had empty blister pack available/missing, but reported taking ALu at a incorrect time was taken as probably non adherent.

#### ***Adherent patients***

A patient with empty blister pack available/missing, and reported taking all six doses of ALu at a correct dose and correct time was categorized as probably adherent. Those with an empty blister pack available but no tablet remaining, and reported taking all six doses of ALu at a correct dose and correct time was categorized as definite adherent.

### Temperature measurements

Baseline body temperature was taken before treatment and on day 3 and 7. The axillary temperature (a good correlate of core temperature) was taken by using a calibrated digital clinical thermometer. Fever was defined as any elevation of body temperature to ≥37.5°C.

### Microscopy for parasite counts

Blood for slides were taken by a finger prick and processed with Giemsa as required, then microscopy for parasite count was done at the dispensary. Microscopy and measurement of axillary temperature was done by a trained research assistant, who was also a member of the field team.

### Day-7 follow up visit

Sampling of blood for determination of lumefantrine plasma concentration was carried out at the dispensary on day-7. Day-7 corresponds to 24 hours after 7 days of ALu intake. Axillary temperature measurements and microscopy for malaria parasites count were also done. A single venous blood sample (2 ml) was collected from each eligible participant into sodium heparinized vacutainers. Each vacutainer was labelled appropriately with subject’s identification number (ID) and date. Plasma samples for determination of lumefantrine plasma concentrations were obtained by centrifugation within 30 minutes after collection of blood samples using centrifuge operated by a generator. The plasma samples were transferred into labelled cryovials and immediately stored in liquid nitrogen at -190°C. The samples were transported in liquid nitrogen to MUHAS for further storage at -80°C and analysis.

### Bioanalytics

Plasma samples were analysed at MUHAS-Sida Bioanalytical laboratory located at Muhimbili University of Health and Allied sciences. The plasma analysis for blood lumefantrine determination was done using an HPLC method with UV detection [16].

### Ethical considerations

Ethical clearance was granted by Muhimbili University of Health and Allied Sciences (MUHAS) Ethical Committee and permission to carry out the study was obtained from the District Executive Director (DED), DMO and village leaders. Participants were asked to consent/assent for participation and were free to refuse participating in the study.

### Data analysis

Analysis was done using SPSS version 16.0 (SPSS, Inc., Chicago, IL) software. Categorical variables were analysed using Chi-square statistical test. The categories of adherence were presented as proportions and compared among age groups using a chi-square test. Association between adherence and several exposure variables (age, educational level of the respondent, occupation of the respondent, family size, number of children cared for by the respondent, place where the first dose was taken, presence/absence of fever on presentation, parasitaemia on presentation) were first analysed in a univariate model using a Chi-square test. The independent variables associated with adherence at the *P* < 0.5 levels were entered in a multivariate logistic regression model. Lumefantrine plasma concentrations were described by age group and category of adherence as means with corresponding standard deviations. First the lumefantrine concentration was described with reference to age and the cut-off value of 175 ng/ml. Using the ANOVA statistics the mean lumefantrine concentrations were compared in different age groups, and then with the level of adherence. The results were regarded as statistically significant if the p-value was < 0.05. Finally the agreement between verbal and pill-count responses on drug intake were validated by kappa coefficients.

## Results

### General characteristics of the participants

**Figure 2:**
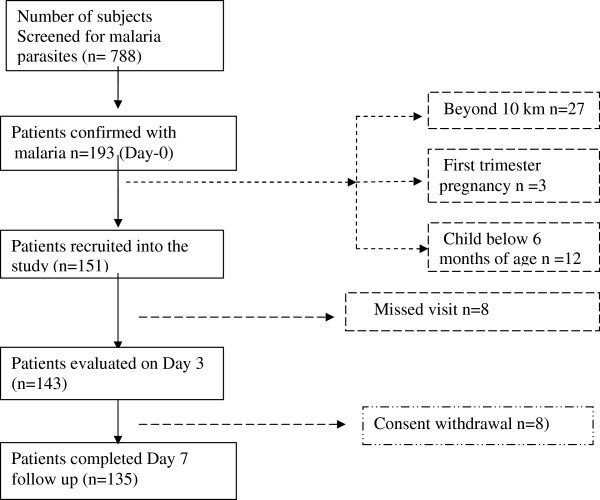
Number of patients traced and interviewed and those not traced.

Table 1 describes the baseline information and demographic characteristics of the studied patients. A total of 788 patients were screened for malaria, of whom 193 (24.49%) were confirmed with malaria parasites. Among the malaria patients, 151 met the inclusion criteria so were enrolled into the study. Among the 151 patients, 122 were children (0–17 years) and the rest were adults (18+ years).Eight patients could not be traced on day-3, moreover eight patients did not return to the dispensary on day-7 for venous blood sampling making a total of 16 patients lost-to-follow-up. Day-7 data was available in 135 patients. Figure 2 summarizes the recruitment pattern and 135 patients completed the study.

**Table 1:** Baseline information and demographic characteristics of participants

**Age group (years)**		**0-5**	**6-12**	**13-17**	**18+**	**Total**		**p value**
Inclusion n = 151 (day 0)								
Fever (≥37.5°C)	Yes	30	22	1	7	60	61.2%	<0.001
	No	5	12	6	15	38	38.8%	
Parasitaemia (/μl)	<2000	3	5	2	13	23	15.2%	<0.05
	2000-100000	46	41	10	16	113	74.8%	
	>100000	11	4			15	9.9%	
Home visit (day 3) n = 143								
Sex of respondent	Male	2	14	6	13	35	24.5%	<0.001
	Female	54	34	6	14	108	75.5%	
Education level of respondent	At least primary	29	28	10	24	91	63.6%	<0.01
	No formal	27	20	2	3	52	36.4%	

### Self-reported adherence

Among 143 patients who could be traced at home on day 3, 114 (79.7%) reported to have finished all the tablets dispensed from the dispensary. Among those who finished the drug, 104 (91.2%) reported that they took the drug at correct dose but incorrect time (outside ±4 hours deviation from dose 3–6) and thus they were defined as probably non-adherent. Only 10 (8.8%) of the patients took all the doses at the correct dose and time and hence were classified as probably adherent. The overall adherence was at 10/143 (7%) level. Among all patients interviewed, 29/143 (20.3%) admitted to have missed ≥1 doses of ALu. The level of reported non-adherence was highest (50%) in the age group 13–17 as compared to other age groups and lowest (7.4%) in the 18+ years group. The difference in the level of reported tablet consumption was statistically significant in the different age groups (*p* = 0.024) (Table 2).

**Table 2:** Reported Tablet Consumption

	**Patient age (years)**				
**Reported tablet consumption**	<5* (n = 56)	6-12* (n = 48)	13-17* (n = 12)	**18+ (n = 27)**	**Total (n = 143)**
Finished dose	44 (78.6%)	39 (81.2%)	6 (50.0%)	25 (92.6%)	114 (79.7%)
Not finished	12 (21.4%)	9 (18.8%)	6 (50.0%)	2 (7.4%)	29 (20.3%)

*The respondent was the mother or guardian of the child of the mentioned age.

### Blister packs check and pill count

Blister packs were available in 122 (85.3%) among 143 patients found at home on day-3 during uninformed visit of the households. Tablets were still remaining on day-3 in 29/122 (23.8%) of the patients who could show the blister packs, thus classified as definitely non-adherent. Among the patients with tablets remaining, 21/29 (72.4%) had 1–4 tablets, 6/29 (20.7%) had 5–8, 1 (3.4%) had 12 and 1 (3.4%) had 20 tablets remaining. No blister packs could be shown by 21 (14.7%) of the patients.

### Tablets remaining per level of education of the patient or caregiver

In assessing the number of tablets remaining by level of education of patients/caretakers (those with no formal education and those with at least a primary school education) it was shown that, education did not influence the extent of adherence (p = 0.239).

### Overall adherence after combining self-report method and pill count

The results from self reported responses and blister packs check were combined and analysed together using the three categorical variables. The results are summarized in Table 3.

**Table 3:** Overall adherence after combining self-report and pill count methods

	**Age of participants (yrs)**				
**Adherence**	**≤12 (n = 104)**	**13-17 (n = 12)**	**≥18 (n = 27)**	**Total (n = 143)**	***p-value***
Probably adherent	8 (7.7%	0	2 (7.4%)	10 (7.0%)	0.044
Probably non adherent	75 (72.1%)	6 (50%)	23 (85.2%)	104 (72.7%)	
Definitely non adherent	21 (20.2%)	6 (50.0%)	2 (7.4%)	29 (20.3%)	

### Agreement between self-report method and pill count

Kappa coefficient was determined and used to test the agreement between the self-reported responses on drug intake and pill-counts. Of the responses given, 95.5% were concordant (the patient reported finishing the treatment course and there were no tablets left in the blister pack, or the patient reported not finishing the treatment and there were tablets remaining in the blister pack). The kappa coefficient, for this population was 0.955 (very close to 1) indicating strong agreement.

### Timing of ALu tablet intake

**Figure 3:**
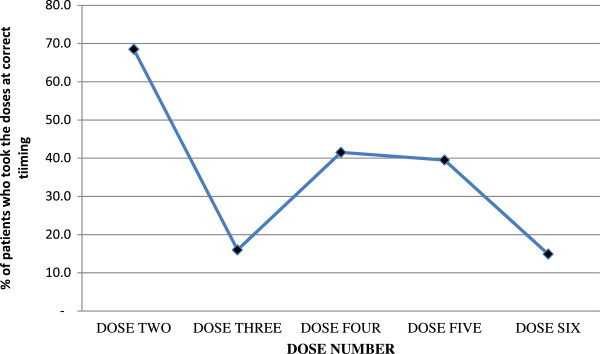
Correct timing pattern for ALu intake across the doses.

Figure 3 illustrates the trends of correct timing for each dose taken by patients. Most patients, 98 (68.5%) took their first dose at home while the rest had taken their first dose while still at the dispensary. Regarding the intake of specific doses, 110 (76.9%) reported to have taken the second dose at the correct dose and time (First day, 8 ± 1 hours after the first dose).

Regarding dose 3, only 22/137 (16.0%) patients reported to have taken the dose at the correct dose and time (24 ± 4 hours after dose one). Regarding the off-schedule doses, the majority of the patients, 86/137 (62.8%) had taken the drug 8 hours after the second dose i.e. 16 hours after the first dose. Among the rest, 26 (19.0%) took the drug after 12 hours and three (2.2%) patients could not remember the time they took the third dose.

Regarding the intake of the fourth dose, 56/135 (41.5%) patients reported to take ALu at the correct dose and time (Second day, 12 ± 4 hours after the third dose), and the rest (58.5%) took the drug at a wrong time and eight patients did not the second dose.

The fifth dose was taken correctly (12 ± 4 hours after dose 4) in 51/129 (39.5%) of the patients. The rest could not take the dug at the correct time. Moreover, 14 patients did not manage to take this dose.

Dose six (last dose) was taken correctly (12 ± 4 hours after dose 5) by 17/114 (14.9%) of the patients whereas the rest took the drug at incorrect time. Among the patients with off-schedule dosing, 92/114 (80.7%) did not take the last dose on time and 5/114 (4.4%) could not remember the time they took the last dose. This dose was not taken at all by 29 patients.

The overall trend of timing of drug intake indicates that dose 2 was taken correctly in most of the patients and dose 3 was taken correctly compared to dose 4 and 5. The last dose was taken correctly though by the least number of patients. The influence of the place where first dose was taken on adherence was observed. Those who took their first dose at the dispensary had a higher chance of adherence to treatment regimen as compared to those started at home (p = 0.007).

### Fatty meal requirements and vomiting

Regarding the method of ALu intake, the majority of patients, 137/143 (95.8%) took the drug with water alone while the rest took the drug either alone or with soft beverages. During the whole course of treatment, 21/143 (14.7%) reported to have vomited one or more doses of the drug. They were asked on which action to take if vomited one or more of the doses and the majority, 55 (38.5%) could not tell about what action to take. However the difference in the actions to be taken by the participants in the event vomiting occurs was not statistically significant in the different age groups (*p* = 0.405).

### Predictors of adherence

Various explanatory (independent) variables, such as age and sex of the patient, Day-0 temperature, day-0 parasites counts, education, occupation, marital status of the participant/caretaker, family size, total number of children cared by the same caretaker and place where first dose was taken were analysed by univariate *t*-test to find if they were significantly associated with overall adherence. Only four factors, i.e. age, sex, education and place where first dose was taken, were associated with the adherence at p < 0.1. Age group 0–12 years was negatively associated with adherence at p = 0.026. Female gender was negatively associated with adherence at p = 0.064. Primary education was associated with the outcome at p = 0.090. Taking the first dose at home was negatively associated with adherence at p = 0.014. When these four factors were fitted into a multiple logistic regression model, only place where first dose was taken remained significantly associated with adherence at p < 0.005. With this last variable, the Odds of becoming adherent if first dose was taken at home was 0.025 while the Odds of becoming adherent if first dose taken at the dispensary was 0.22. With this respect the Odds ratio was 0.22/0.025 = 9.6, implying that those who took the first dose at the dispensary were 9.6 times more likely to adhere as compared to those who took the first dose at home.

### Blood lumefantrine concentration

Only 127/135 (94.07%) patients who provided blood for Day-7 lumefantrine plasma concentration determination had analyzable samples. The plasma samples for eight patients were not analysable due to low plasma volume and some had haemolysed blood. Sixteen patients were excluded from analysis due to vomiting of one or more doses during the course of treatment. Lastly, 21 patients who were definitely non-adherent (by pill count) were excluded from lumefantrine level data analysis. Therefore, blood samples from only 90 patients could be analysed for lumefantrine plasma concentrations. Patients with lumefantrine concentration <50 ng/ml were assigned a zero value. Among 90 patients 13/90 (14.4%) had lumefantrine concentration <175 ng/ml, while the rest had ≥175 ng/ml. The mean lumefantrine concentration in the adherent patients (by self-report and pill count) was higher than in the non-adherent patients (p = 0.643).

The mean lumefantrine concentration was lowest in the 0–5 years age group (365.65 ng/ml, 95% CI = 259.32-471.97) as compared to other age groups; 6–12 (450.61 ng/ml, 95% CI = 364.96-536.26), age group 13–17 (395.58 ng/ml, 95% CI = 54.01-737.14) and in the 18+ age group, (778.89 ng/ml, 95% CI = 525.17-1032.60) (p < 0.001). When subjected to *post hoc* analysis the mean difference was statistically significant in between age groups 0–5 yrs and 18+ (p < 0.001) and between age group 6–12 and 18+ (p = 0.007).

The mean lumefantrine concentration for the patients who took their first dose at the dispensary was lower, (473.43 ng/ml, 95% CI =365.83-581.02) as compared to those who took the first dose from homes, (507.38 ng/ml 95% CI =398.44-616.32). However, the observed difference was not statistically significant (p = 0.697).

The median lumefantrine concentration was higher in the adherent group (586.20 ng/ml, range 268.60-715.90) as compared to non-adherent group (403.20 ng/ml range 0.00-2239.80). The overall median lumefantrine concentration was 442.40 ng/ml (range 0–2239.80 ng/ml). When compared in different age groups, the median lumefantrine concentration was significantly lower in the younger age group i.e. 0–5 year (296.35 ng/ml, range 1213.70) as compared to age group 6–12 (440.30 ng/ml, range 1200.60), age group 13-17 (388.45 ng/ml, range 525.40) and the age group 18+ (641.40 ng/ml, range 2239.80) [p < 0.001].

### Parasite counts on day-0, day-3 and day-7

Baseline parasitaemia is shown in Table 2. However, no parasites were detected on day-3 and day-7 after drug administration in all patients regardless of the adherence status and fever subsided in all patients.

## Discussion

This study has demonstrated a relatively low adherence to ALu treatment regimen in a rural community in Tanzania, more than six years after ALu had been introduced as first line drug of choice for treatment of non-severe falciparum malaria. The study has shown low adherence to ALu, since only 10/143 (7.0%) of the participants took all six doses at correct dosage and time. These findings disagree with what Kabanywani *et al.* and Simba *et al.* reports [7,10]. In these studies, the time of ALu intake was also considered but the obtained adherence rate was high. The study currently reported was conducted six years after switching from SP to ALu. A new policy is normally accompanied with community sensitization, nascent training of health workers and high availability of medication both of which are likely to positively influence the extent of initial adherence to treatment. The Ugandan study which assessed adherence to ALu after the drug had just been introduced in the country obtained high adherence results confirming the role played by the above mentioned strategies [5].

Despite the differences in adherence rates obtained by the use of different methods, no detectable parasite was observed by microscopy and fever subsided in all patients (Table 1). There was no detectable Day-3 and Day-7 parasitaemia in all patients even those who were not-adherent including the ones with low lumefantrine plasma concentrations indicating that plasma concentration of an anti-malarial drug is not the only factor that determines parasite clearance. Extensive parasite clearance may further be explained by the mode of action of artemether and lumefantrine drug combination. Artemether provides a rapid reduction of parasitaemia by over 90% within 24 hours of treatment and almost complete eradication after 36 hours [17]. Furthermore, artemether is absorbed rapidly and biotransformed to dihydroartemisinin and both parent drug and the metabolite are active. Lumefantrine has a slow onset and acts slowly eliminating the residual parasites [17,18]. Nevertheless, it should be borne in mind that, the total parasite clearance is dependent on many factors including parasite density at baseline [19].

This study applied patient/catetaker self report as measures for adherence (Table 2) and was further subjected to pill count using uninformed home visit in order to get reliable data. Studies have shown that, the use of self-report alone could lead to an overestimated adherence [2]. Uninformed home visit, which was accompanied with pill count and interview on self-report, observed that the packs were still available in 122/143 (85.3%) of the patients visited at their homes. Agreement between adherence obtained by patient self-report and pill count demonstrated that the two methods were complementary in this population (Kappa coefficient = 0.955). The overall adherence of after combining pill count and self-report is reflected in Table 3.

To further ascertain the adherence obtained by self report and pill count, day-7 lumefantrine plasma concentration was also used as supplementary marker for assessing adherence to ALu intake (Table 4). Lumefantrine plasma concentration of ≥175 ng/ml has been reported to be a good predictor of malaria cure for adhering patients [2,17]. In the present study, day-7 lumefantrine plasma concentration was inadequate (<175 ng/ml) in 14.4% of the studied patients (Table 4). This means, these patients would be at a risk of therapeutic failure or recrudescence [14]. Low day-7 plasma concentrations observed in these patients could have been attributed by poor adherence, erratic absorption or low metabolizing capacity of the drug. Recently, high inter-individual variability (>60%) in the plasma concentrations of lumefantrine was observed in healthy individuals who had taken the drug with fatty meal and under supervision [20]. Lower mean lumefantrine concentration in the younger age group (0-5 yrs) as compared to other age groups (6-18+ yrs) was observed (Table 4). These findings are in agreement with those reported by the Mbarara study in Uganda [5]. Low lumefantrine concentration in this age group may be due to vomiting the drug by some children. It is also possible that, some caregivers forgot to give the medications to their children on time.

**Table 4:** Mean lumefantrine concentration -patient age

	**Patient age**					***p-value***
**Lumefantrine concentration**	**0-5 (n = 30)**	**6-12 (n = 35)**	**13-17 (n = 4)**	**18+ (n = 21)**	**Total (n = 90)**	
<175 ng/ml	7 (23.3%)	5 (14.3%)	1 (25.0%)	0 (0.0%)	13 (14.4%)	0.1204
≥175 ng/ml	23 (76.7%)	30 (85.7%)	3 (75.0%)	21 (100.0%)	77 (85.6%)	

In this study the majority of patients (68.5%) preferred to take the first dose at home. The main reason given was the lack of water and/or food required for ALu intake at the dispensary and therefore majority initiated the dosing at home. It can be seen that about 40% of patients missed the correct timing of the second dose and a worse scenario is observed in dose 3 and the last dose (Figure 3). However, there was higher adherence with regard to timing of the second dose and the overall adherence in those patients who took their first dose at the dispensary was relatively higher as compared to those who initiated the treatment at home (p = 0.007). The Tanzanian National Guidelines for Diagnosis and Treatment of Malaria recommends initiation of first dose of ALu at the dispensary [4]. This provides opportunity to receive adequate instructions on the proper timing for taking the subsequent doses at home.

The present study has shown that education level had no significant influence on the extent of adherence in the rural population. There was no statistically significant difference between those who had no formal education and patients with at least primary education (*p =* 0.072).However it is important to note that, those who were definitely non-adherent were only 20.3% implying that, it is the component of those who were categorized as probably non-adherent (72.7%) that affected the overall adherence rate in this study. The low adherence rate hereby reported is mainly attributed by taking the tablets at an incorrect time interval (Figure 3) and not because of unfinished tablets (required time ± 4 hrs).

The limitation of this study is a recall bias by some patients on the actual time for taking each dose in line with the dosage regimen. Lack of genetic profiles from the study population with regard to variation in drug metabolism among individuals is also a limitation to this study.

## Conclusion

Adherence to treatment with artemether-lumefantrine drug combination six years after the change of malaria treatment policy declined compared to what was reported at the point of policy introduction. The mainly reason that contributed to low adherence rate was due to poor timing of intake of the subsequent doses. Continuous monitoring of the extent of adherence to treatment is essential in ensuring the *status quo*. This is important in instituting corrective measures without delay. It is important to continuously provide adequate adherence counseling to patients on the importance of taking the doses at the recommended time interval.

## Competing interests

The authors declare that they have no competing interests.

## Authors’ contribution

OMSM and SP conceived the study, and participated in its design, coordination, data analysis and writing of the manuscript. BN participated in the data analysis as well as manuscript preparation. SM participated in study design, data collection and analysis and contributed in the preparation and writting of the manuscript. All authors read and approved the final manuscript.
